# Body composition predicts hypertension using machine learning methods: a cohort study

**DOI:** 10.1038/s41598-023-34127-6

**Published:** 2023-04-27

**Authors:** Mohammad Ali Nematollahi, Soodeh Jahangiri, Arefeh Asadollahi, Maryam Salimi, Azizallah Dehghan, Mina Mashayekh, Mohamad Roshanzamir, Ghazal Gholamabbas, Roohallah Alizadehsani, Mehdi Bazrafshan, Hanieh Bazrafshan, Hamed Bazrafshan drissi, Sheikh Mohammed Shariful Islam

**Affiliations:** 1 Department of Computer Sciences, Fasa University, Fasa, Iran; 2 Student Research Committee, Shiraz University of Medical Sciences, Shiraz, Iran; 3 Non Communicable Diseases Research Center, Fasa University of Medical Sciences, Fasa, Iran; 4 Bone and Joint Diseases Research Center, Department of Orthopedic Surgery, Shiraz University of Medical Science, Shiraz, Iran; 5 Department of Computer Engineering, Faculty of Engineering, Fasa University, Fasa, 74617-81189 Iran; 6 Institute for Intelligent Systems Research and Innovation (IISRI), Deakin University, Geelong, Australia; 7 Shiraz University of Medical Sciences, Shiraz, Iran; 8 Department of Neurology, Clinical Neurology Research Center, Shiraz University of Medical Sciences, Shiraz, Iran; 9 Cardiovascular Research Center, Shiraz University of Medical Sciences, PO Box: 71348-14336, Shiraz, Iran; 10 Institute for Physical Activity and Nutrition, School of Exercise and Nutrition Sciences, Deakin University, Geelong, VIC Australia; 11 Cardiovascular Division, The George Institute for Global Health, Newtown, Australia; 12 Sydney Medical School, University of Sydney, Camperdown, Australia

**Keywords:** Computational biology and bioinformatics, Diseases

## Abstract

We used machine learning methods to investigate if body composition indices predict hypertension. Data from a cohort study was used, and 4663 records were included (2156 were male, 1099 with hypertension, with the age range of 35–70 years old). Body composition analysis was done using bioelectrical impedance analysis (BIA); weight, basal metabolic rate, total and regional fat percentage (FATP), and total and regional fat-free mass (FFM) were measured. We used machine learning methods such as Support Vector Classifier, Decision Tree, Stochastic Gradient Descend Classifier, Logistic Regression, Gaussian Naïve Bayes, K-Nearest Neighbor, Multi-Layer Perceptron, Random Forest, Gradient Boosting, Histogram-based Gradient Boosting, Bagging, Extra Tree, Ada Boost, Voting, and Stacking to classify the investigated cases and find the most relevant features to hypertension. FATP, AFFM, BMR, FFM, TRFFM, AFATP, LFATP, and older age were the top features in hypertension prediction. Arm FFM, basal metabolic rate, total FFM, Trunk FFM, leg FFM, and male gender were inversely associated with hypertension, but total FATP, arm FATP, leg FATP, older age, trunk FATP, and female gender were directly associated with hypertension. AutoMLP, stacking and voting methods had the best performance for hypertension prediction achieving an accuracy rate of 90%, 84% and 83%, respectively. By using machine learning methods, we found that BIA-derived body composition indices predict hypertension with acceptable accuracy.

## Introduction

Hypertension is one of the most important and preventable causes of cardiovascular disease (CVD), stroke, chronic kidney disease, and dementia which caused approximately 8.5 million deaths in 2015, in low & middle-income countries^1^. Reports show that hypertension prevalence is 25% in Iran^2^. Hypertension depends on well-known risk factors such as age, gender, family history, smoking, alcohol consumption, central obesity, overweight and physical inactivity^3,4^. Obesity has gained significant attention over the past years^5^.

Body mass index (BMI) is widely used for anthropometric measurements, and regardless of inaccuracy, it is still commonly used to determine obesity and assess health risks such as hypertension^6^. Complementary measures such as waist circumference, waist-to-hip ratio (WHR), and body composition analysis improve the prognostic efficiency of BMI^7^. Evidence shows that body fat distribution is a more vital determinant of cardiovascular morbidity and mortality than increased fat mass^8–10^; further indicating that detailed assessment of body composition is beneficial for health risk estimations.

In the past few years, growing number of researchers have used machine learning and data mining algorithms to diagnose and treat health conditions such as heart^11^ and brain^12^ diseases. Their non-invasive nature and accuracy have enabled health professionals to quickly identify at-risk individuals and use more efficient preventive and managing strategies^13^.

In this study, we used machine learning approaches to investigate whether BIA-derived body composition indices predict hypertension in a cohort of patients.

## Methods

### Study design and participants

Fasa cohort study^14^ recruited at least 10,000 people and assessed predisposing factors for non-communicable diseases in rural regions of Fasa, Iran. In the present study, we used a subset of their data of 4663 records in which 2156 were male, 1099 had HTN, and the age range was 35–70. hypertension diagnosis was based on the blood pressure threshold defined by ACC/AHA guidelines^15^. All participants had given informed consent, and the Shiraz University of Medical Sciences ethics committee approved this study.

### Body composition analysis

Body composition analysis was performed using eight electrodes (Tanita Segmental Body Composition Analyzer BC-418 MA Tanita Corp, Japan) BIA machines. The following variables were measured:

1. Fat mass (FATM): Total fat mass (FATM), Left and Right Leg Fat Mass (LLFATM & RLFATM), Left and Right Arm Fat Mass (LAFATM & RAFATM), and Trunk Fat Mass (TRFATM).
2. Fat percentage (FATP): Total Fat Percentage (TFATP), Left and Right Leg Fat Percentage (LLFATP & RLFATP), Left and Right Arm Fat Percentage (LAFATP & RAFATP), Trunk Fat Percentage (TRFATP).

Fat percentage is calculated as (fat mass)/weight × 100

3. Fat-free mass (FFM): Total Fat-Free Mass (FFM), Left and Right Leg Fat-Free Mass (LLFFM & RLFFM), Left and Right Arm Fat-Free Mass (LAFFM & RAFFM), Trunk Fat-free Mass (TRFFM).
4. Basal metabolic rate (BMR).

### Dataset

Our dataset included 4663 records of which 1099 were hypertensive. Among 2156 males and 2507 females, 430 and 669 cases were hypertensive, respectively. Input features were: age (Between 35 and 70), gender ID (1: male, 2: female), BMR, FATM, FATP, FFM, LLFATP, RLFATP, LLFFM, RLFFM, LLFATM, RLFATM, LAFATP, RAFATP, LAFATM, RAFATM, LAFFM, RAFFM, TRFATP, TRFATM, and TRFFM. The target feature is the discrete binary variable of hypertension i.e. Yes or No.

It is noted that Institutional approval was granted for the use of the patient datasets in research studies for diagnostic and therapeutic purposes. Approval was granted on the grounds of existing datasets. Informed consent was obtained from all of the patients in this study. All methods were carried out in accordance with relevant guidelines and regulations. Ethical approval for the use of these data was obtained from the Tehran Omid hospital.

### Investigated machine learning and data mining algorithms

We utilized some of the most efficient classification algorithms, such as Support Vector Classifier (SVC)^16^, Decision Tree (DT)^17^, Stochastic Gradient Descend (SGD) Classifier^18^, Logistic Regression (LR)^19^, Gaussian Naïve Bayes (GNB)^20^, K-Nearest Neighbor (K-NN)^21^, Multi-Layer Perceptron (MLP)^22^, Random Forest (RF)^23^, Gradient Boosting (GB)^24^, Histogram-based Gradient Boosting (HGB)^25^, Bagging^26^, Extra Tree (ET)^27^, Ada Boost^28^, Voting^29^, and Stacking^30^.

These algorithms are briefly explained, and references for more detailed descriptions about them are provided. In the following part, we introduce metrics for evaluating the effectiveness of the algorithms.

To classify the data, SVC tries to find the best hyperplane to separate the different classes. The criterion to evaluate the hyper-plane is maximizing its distance to the sample points. SVC has a limitation compensated by the Support Vector Machine (SVM) non-linearly. It is the difference between SVC and SVM. In SVC, the hyper-plane classifies the data linearly. However, in SVM, the algorithm separates the dataset non-linearly^31^.

DT is a supervised learning algorithm used for classification and regression. This method tries to learn a model that can predict the value of a target feature by learning some decision rules inferred from the features of samples^32^.

SGD classifier is a linear classifier optimized by the SGD^33^.

LR is a classification algorithm used in machine learning; it uses a logistic function to model the dependent variable. This variable can only have two values. Therefore, LR is only used in solving problems with binary target features. Moreover, the sigmoid function in LR maps the predicted values to the probabilities^34^.

GNB is a probabilistic classification algorithm that utilizes the Bayes theorem. It assumes that the variables are independent of each other. This algorithm requires training data to estimate the parameters needed for classification. Since its implementation is simple, it is used to solve many classification problems^20^.

K-NN algorithm is a non-parametric, supervised classifier that uses proximity to perform classification. In this algorithm, the assumption is that similar points are located near each other. A class label is assigned to a sample based on the majority vote between its K nearer samples^35^.

MLP is a supervised learning algorithm that tries to learn a function based on a data set. The learned function is used to predict the class for a new sample. This algorithm has a network structure consisting of several layers of nodes. Each layer is connected to the next layer in the network. Nodes in the first layer represent input data. Other nodes map inputs to outputs by linearly combining them using a set of weights and a bias and applying an activation function^36^.

RF is an ensemble learning method for classification which consists of many decision trees. It is created based on training data. The output of this algorithm is the class that most trees suggest. This algorithm can be used to avoid over-fitting the training set. Random forest performance is usually better than decision tree classifiers. However, the performance improvement usually depends on the data type^37^.

Another machine learning algorithm is GB which performs prediction based on a set of weak prediction models such as decision trees. GB is one of the most popular methods of structured classification and predictive regression modeling and can cover a wide range of data sets. However, this method suffers slow training, mainly when used on large data sets (number of samples ≥ 10,000). In order to solve this problem, the trees added to the set are trained by discretization (binning) of continuous input variables to hundreds of unique values^24^. This modification dramatically increases the algorithm execution speed compared to the Gradient Boosting Classifier. GB ensembles that implement this technique are referred to as HGB sets. It can also manage missing values. During training, at each split point, the tree learns whether samples with missing values should be assigned to the left or right child based on the potential gain. If there are no missing values for a given feature during training, samples with missing values are mapped to the child that has the highest number of samples^25^.

A bagging classifier is an ensemble meta-classifier that consists of a set of base classifiers applied to random subsets of the original dataset. These classifiers’ results are collected, and a final prediction is derived according to them. The base classifiers are trained in parallel on disjoint training sets. Much of the original data may be repeated in the resulting training set, while other data may be omitted^38^.

ET classifier is an ensemble learning technique, also known as an extremely randomized tree classifier. This algorithm uses the results of several uncorrelated decision trees collected in a forest to perform the classification process. The performance of this algorithm is very similar to an RF classification. However, building decision trees in the forest is different from RF. In this algorithm, each decision tree is built from the original training sample. At each test node, each tree is presented with a random sample containing a subset of the feature set. Each decision tree must select the best feature for splitting the data based on mathematical criteria such as the Gini index. Random selection of samples leads to multiple uncorrelated decision trees^27^.

An Adaptive Boosting or Adaboost classifier is a meta-classifier algorithm. This ensemble algorithm starts by fitting a classifier on the original data set. It then tries to classify the same data set again using additional copies of the classifier, except that the weights of the misclassified samples are adjusted so that subsequent classifiers focus more on complex cases. The outputs of these classifiers are combined using weighted summation to create the final classification output^39^.

The voting classifier is a meta-classifier that trains base models the outputs of which are used to guess the final result. Aggregation of the results of base learners is done in two ways: hard voting and soft voting. In the former, voting is done based on the output class declared by each base learner, while in the latter, the output class is based on the probability predicted by the base classes^40^.

Stacking or Stacked Generalization is an ensemble meta-learning algorithm. Using this algorithm makes it possible to learn how to combine the results of two or more basic machine learning algorithms in the best possible way. The advantage is that capabilities of a wide range of well-performing algorithms can be exploited to achieve performance that none of them can achieve individually^41^.

We will apply these algorithms to our dataset but before that, some preprocesses must be performed on the training data.

### Data preprocessing

To improve the performance of algorithms, some feature selection algorithms were used. These algorithms are used for selecting a subset of features for model construction. They are commonly used for simplification of constructed models to make them easier to interpret. Using these techniques leads to shorten training time, and void the curse of dimensionality. The feature selection algorithms tested in our research are best first^42^, genetic algorithm^43^, greedy forward selection^44^, greedy backward elimination^44^, decision tree^45^, random forest^46^, and particle swarm optimization (PSO)^47^. Among them, genetic algorithm showed the best performance and the rest of this research was organized according to the results of it. This algorithm declared that FATP, AFFM, BMR, FFM, TRFFM, AFATP, LFATP, and older age were the top features in hypertension prediction.

### Evaluation metrics

**Figure 1:**
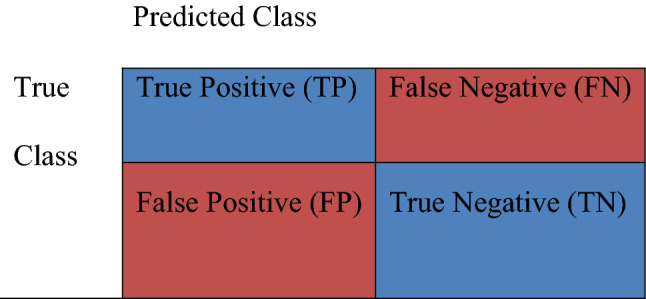
Confusion matrix and its data.

In this research, we used the confusion matrix to test and compare the algorithms’ effectiveness. This matrix is a popular metric to evaluate the performance of binary and multi-class classification problems. Figure 1 shows a confusion matrix^48–50^.

The confusion matrix shows how many outputs are correctly classified and how many are misclassified. In this table, “TN”, for true negative, shows how many negative samples are correctly classified. Similarly, “TP” stands for true positive and indicates how many positive samples are correctly classified. The term "FP" stands for false positive and represents the number of samples misclassified as positive. Finally, "FN" stands for false negative and indicates the number of positive samples misclassified as negative. Based on the values of this matrix, one of the most common metrics used for evaluating classification algorithms –accuracy- is calculated based on Eq. (1)^51,52^.

1 $$Accuracy=\frac{TP+TN}{TP+TN+FP+FN}.$$

Precision, sensitivity (or recall), specificity, and F1-score are some other performance metrics that are very popular. They are calculated according to the following equations:

2 $$Macro\,\, Average\,\, Precision=\frac{\frac{TP}{TP+FP}+\frac{TN}{TN+FN}}{2},$$

3 $$Macro\,\, Average \,\,Sensitivity = \frac{\frac{TP}{TP+FN}+\frac{TN}{TN+FP}}{2},$$

4 $$Specificity=\frac{TN}{TN+FP},$$

5 $$F1-score=2*\frac{precision*sensitivity }{precision+sensitivity}.$$

**Figure 2:**
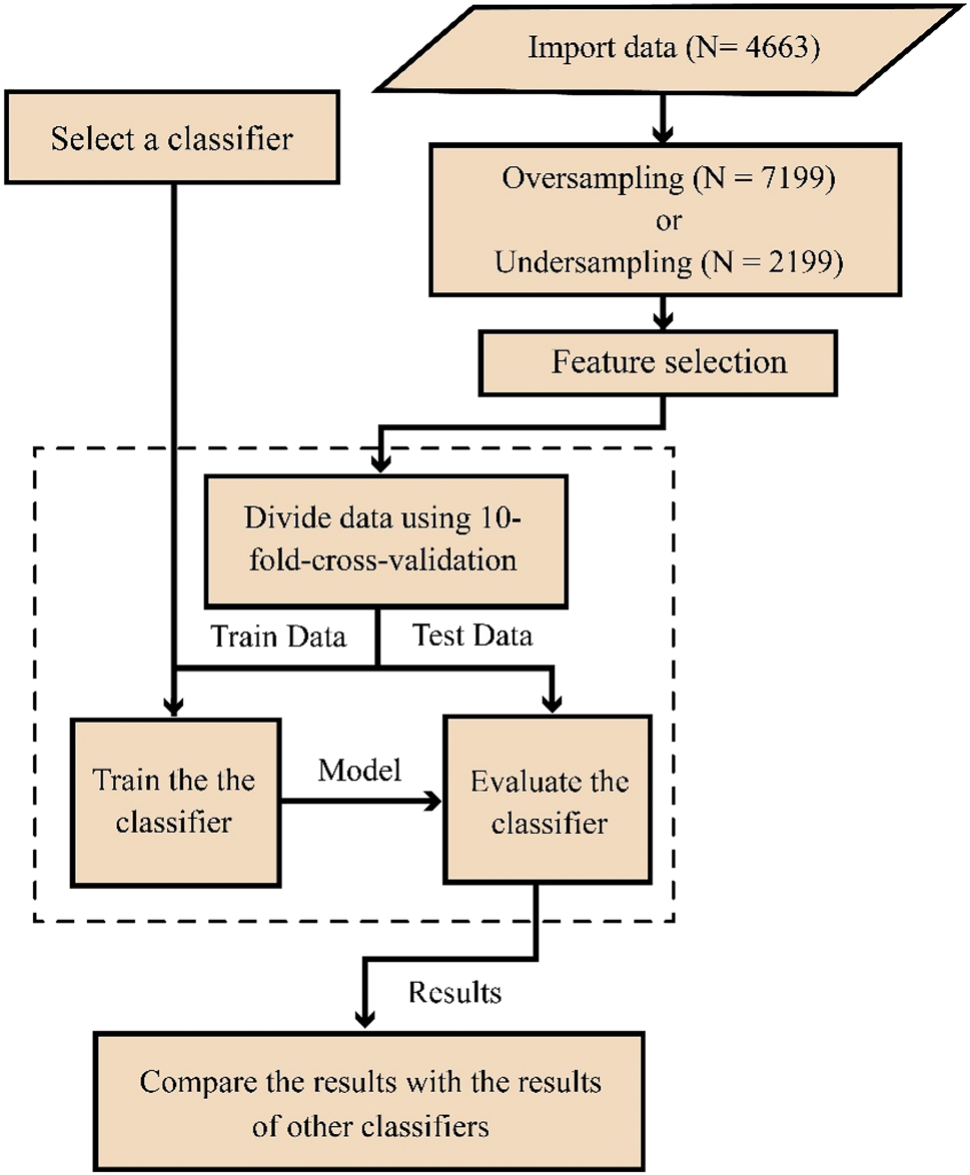
The flowchart of the methodology used in this research.

Using these metrics, the above mentioned classification algorithms are compared. The flowchart of proposed method is shown in Fig. 2.

As there is an obvious category imbalance between normal individuals (negative cases) and diseased individuals (positive cases), during model training, the prediction results may be biased to judge as normal individuals, resulting in high specificity and low sensitivity. To solve this issue, three oversampling and two undersampling methods were applied to the dataset. SMOTE^53^, Random Oversampling^54^, ADASYN^55^ methods are oversampling and Random Undersampling^56^ and NearMiss^57^ methods were used for undersampling. However, the results of applying classification methods on oversampling data generated by SMOTE and undersampling data generated by NearMiss methods were reported because of better performance. Using SMOTE, the number of cases was increased to 7128 with an equal number of positive and negative cases. When NearMiss was used for undersampling, the number of cases was decreased to 2198 with equal number of samples in each class.

In addition, the MetaCost^58^ method was used to increase the penalty of negative cases.

## Experimental results

In this section, we report and compare the results of applying classification algorithms mentioned in the methodology section. These algorithms are implemented in Python version 3.10,0 and its ready-made modules were used. These algorithms were run in Windows 11 operating system. The default settings of the algorithms are used in this research, except those listed in Table 1.

**Table 1:** Settings of the used algorithms.

Algorithm	Settings
Naïve Bayes	–
SVC	Kernel: linear, random state: 0
DT	Criterion: entropy, maximum depth: 3
SGD	Estimator: SVM
LR	Maximum iteration: 500
GNB	–
K-NN-4	Number of neighbors: 4
RF	–
GB	–
Bagging	Base estimator: decision tree
ET	Maximum depth: 4, number of estimators: 20, random state: 0
Ada boost	Number of estimators: 100
HGB	Maximum iteration: 200
Voting	Estimators: {SVC (kernel: linear, probability: true, random state: 0), gradient boosting, decision tree (criterion: entropy, maximum depth: 3)}, voting: soft
Stacking	Estimators: {SVC (kernel: linear, probability: true, random state: 0), gradient boosting, decision tree (criterion: entropy, maximum depth: 3), extra trees (maximum depth: 4, number of estimators: 20, random state: 0)}
autoMLP	training cycles:10, number of generations: 10, number of esemble mlps: 4

Tables 2, 3, and 4 list the accuracy, precision, recall, f1-score, and AUC of train and test data of these algorithms when oversampling, undersampling, and original data (while the penalty for negative cases in the model was increased) were used, respectively. In our research, genetic algorithm showed the best performance. Therefore, the results reported in Tables 2, 3, and 4 were calculated according to this feature selection algorithm.

**Table 2:** Performance metrics of different classification algorithms applied on oversampled data.

	Algorithm	Accuracy	Macro average precision	Macro average sensitivity	Specificity	AUC
Training phase results	Naïve Bayes	0.76	0.62	0.73	0.79	0.77
	GNB	0.81	0.68	0.80	0.84	0.85
	Bagging	0.83	0.69	0.80	0.84	0.84
	HGB	0.83	0.67	0.80	0.84	0.86
	RNN	0.83	0.66	0.79	0.85	0.81
	SGD	0.84	0.69	0.82	0.85	0.81
	LR	0.84	0.69	0.82	0.89	0.84
	K-NN-4	0.83	0.68	0.81	0.87	0.78
	RF	0.84	0.69	0.82	0.87	0.85
	ET	0.82	0.94	0.80	0.83	0.90
	Adaboost	0.83	0.67	0.81	0.85	0.85
	SVC	0.84	0.82	0.81	0.85	0.84
	DT	0.84	0.74	0.82	0.85	0.87
	GB	0.84	0.73	0.81	0.85	0.88
	Voting	0.85	0.78	0.83	0.89	0.89
	Stacking	0.85	0.78	0.84	0.90	0.91
	autoMLP	0.93	0.90	0.91	0.98	0.92
Testing phase results	Naïve Bayes	0.73	0.60	0.71	0.76	0.72
	GNB	0.80	0.67	0.78	0.84	0.82
	Bagging	0.81	0.68	0.79	0.84	0.82
	HGB	0.82	0.65	0.80	0.83	0.83
	RNN	0.79	0.67	0.77	0.82	0.78
	SGD	0.82	0.67	0.79	0.83	0.79
	LR	0.81	0.69	0.80	0.84	0.83
	K-NN-4	0.81	0.66	0.79	0.84	0.76
	RF	0.82	0.68	0.80	0.84	0.83
	ET	0.83	0.94	0.81	0.85	0.87
	Adaboost	0.82	0.65	0.80	0.86	0.84
	SVC	0.83	0.8	0.80	0.88	0.84
	DT	0.83	0.72	0.81	0.85	0.85
	GB	0.83	0.73	0.81	0.88	0.86
	Voting	0.83	0.76	0.81	0.85	0.88
	Stacking	0.84	0.77	0.82	0.86	0.87
	autoMLP	0.90	0.81	0.89	0.91	0.91

**Table 3:** Performance metrics of different classification algorithms applied on undersampled data.

	Algorithm	Accuracy	Macro average precision	Macro average sensitivity	Specificity	AUC
Training phase results	Naïve Bayes	0.74	0.58	0.69	0.76	0.73
	GNB	0.77	0.65	0.76	0.81	0.82
	RNN	0.77	0.67	0.75	0.80	0.74
	Bagging	0.80	0.66	0.76	0.80	0.81
	HGB	0.79	0.64	0.78	0.81	0.84
	SGD	0.80	0.67	0.79	0.82	0.78
	LR	0.81	0.67	0.79	0.86	0.81
	K-NN-4	0.80	0.65	0.78	0.84	0.75
	RF	0.81	0.66	0.79	0.83	0.82
	ET	0.78	0.91	0.76	0.80	0.86
	Adaboost	0.80	0.64	0.77	0.82	0.82
	SVC	0.81	0.79	0.78	0.81	0.81
	DT	0.81	0.71	0.78	0.82	0.84
	GB	0.81	0.70	0.79	0.82	0.84
	Voting	0.83	0.75	0.80	0.85	0.86
	Stacking	0.82	0.75	0.81	0.88	0.87
	autoMLP	0.90	0.88	0.88	0.94	0.88
Testing phase results	Naïve Bayes	0.71	0.54	0.63	0.72	0.66
	GNB	0.77	0.63	0.75	0.80	0.79
	RNN	0.77	0.65	0.72	0.79	0.71
	Bagging	0.77	0.65	0.76	0.82	0.79
	HGB	0.79	0.63	0.77	0.80	0.81
	SGD	0.78	0.64	0.75	0.81	0.76
	LR	0.79	0.66	0.78	0.82	0.81
	K-NN-4	0.77	0.63	0.77	0.80	0.73
	RF	0.80	0.65	0.76	0.81	0.79
	ET	0.81	0.90	0.78	0.81	0.83
	Adaboost	0.78	0.61	0.77	0.82	0.81
	SVC	0.80	0.77	0.76	0.84	0.81
	DT	0.81	0.70	0.78	0.82	0.81
	GB	0.81	0.69	0.78	0.84	0.83
	Voting	0.80	0.72	0.78	0.82	0.86
	Stacking	0.81	0.75	0.80	0.83	0.83
	autoMLP	0.88	0.77	0.87	0.89	0.88

**Table 4:** Performance metrics of different classification algorithms applied on original data while the penalty for negative cases in the model was increased.

	Algorithm	Accuracy	Macro average precision	Macro average sensitivity	Specificity	AUC
Training phase results	Naïve Bayes	0.72	0.54	0.63	0.73	0.66
	GNB	0.75	0.63	0.74	0.78	0.81
	Bagging	0.78	0.64	0.75	0.79	0.79
	HGB	0.79	0.61	0.74	0.78	0.8
	SGD	0.80	0.64	0.77	0.79	0.77
	LR	0.79	0.64	0.77	0.84	0.79
	RNN	0.76	0.65	0.72	0.80	0.70
	K-NN-4	0.78	0.63	0.76	0.83	0.72
	RF	0.80	0.65	0.78	0.82	0.8
	ET	0.77	0.89	0.75	0.78	0.85
	Adaboost	0.78	0.62	0.75	0.81	0.8
	SVC	0.79	0.76	0.76	0.81	0.8
	DT	0.79	0.69	0.76	0.79	0.82
	GB	0.79	0.67	0.76	0.8	0.83
	Voting	0.80	0.74	0.79	0.83	0.84
	Stacking	0.80	0.73	0.79	0.85	0.85
	autoMLP	0.89	0.86	0.86	0.93	0.88
Testing phase results	Naïve Bayes	0.66	0.51	0.60	0.68	0.61
	GNB	0.76	0.61	0.73	0.78	0.76
	Bagging	0.75	0.63	0.74	0.79	0.76
	HGB	0.77	0.59	0.75	0.77	0.77
	SGD	0.76	0.62	0.74	0.78	0.75
	LR	0.75	0.64	0.75	0.79	0.78
	RNN	0.75	0.64	0.71	0.78	0.68
	K-NN-4	0.76	0.62	0.74	0.8	0.71
	RF	0.76	0.64	0.76	0.78	0.78
	ET	0.78	0.9	0.76	0.8	0.81
	Adaboost	0.77	0.59	0.76	0.8	0.78
	SVC	0.78	0.74	0.75	0.83	0.79
	DT	0.78	0.66	0.77	0.8	0.81
	GB	0.78	0.69	0.77	0.84	0.8
	Voting	0.78	0.71	0.77	0.79	0.83
	Stacking	0.79	0.72	0.76	0.8	0.83
	autoMLP	0.85	0.76	0.85	0.86	0.87

AutoMLP has the best accuracy commonly followed by Stacking and Voting. The performance of different algorithms on the training set was also reported. This helps to check whether the model training is over-fitting or under-fitting, and helps with better adjustment of model parameters to improve the classification results. As it is clear in these tables, the oversampling performance is better than undersampling or original sampling methods.

## Discussion

In the present study and a cohort population, we used machine learning methods and found that BIA-derived body composition indices predict hypertension with an acceptable accuracy. FATP, AFFM, BMR, FFM, TRFFM, AFATP, LFATP, and older age were the top features in hypertension prediction. FATP, AFATP, LFATP, TRFATP, higher age, and female gender directly associated with HTN. But, FFM, AFFM, LFFM, TRFFM, BMR, and male gender were inversely linked to HTN. AutoMLP, stacking and voting methods had the best performance for hypertension prediction showed an accuracy rate of 90%, 84% and 83%, respectively.

### Total FATP and FFM

Various other studies confirm the direct link of body fat mass (and percentage) with blood pressure^59–61^. Park et al.^62^, in a prospective cohort study, showed that a high body fat percentage (more than 19.9% in men and 32.5% in women) was associated with an increased risk of incident hypertension regardless of BMI, waist circumference, and WHR. Although body fat mass and percentage are superior to BMI in morbidities risk assessment, a study^63^ on Iranian population showed that BMI predicts CVD better than body fat percentage. Another study^64^ on American postmenopausal women with normal BMI found no relation between whole-body fat mass and percentage of CVD risk; although regional body fat had significant associations. These discrepancies may be due to different analysis methods of body composition, and ethnicity.

Contrary to our results, some investigations in adult and pediatric populations established that FFM is positively related to systolic, diastolic, or mean blood pressure^65–71^. Korhonen et al.^66^ attribute this finding to muscle mass properties; during daytime and contraction, skeletal muscles release myokines that may increase blood pressure. This explanation confirms the findings of Ye et al.^60^ in a Chinese population: total skeletal mass (TSM) indices -primarily arm lean body mass- are positively associated with blood pressure, pre-HTN, and HTN.

### Trunk FATP and FFM

Previous studies have established the positive association of TRFATM with hypertension and CVD^72^, and our data further support that BIA-measured abdominal adiposity is positively associated with hypertension^73^. Chen et al.^64^ assessed CVD incidence in postmenopausal women with normal BMI during a median of 17.9 years. The authors used Dual X-ray Absorptiometry (DXA) and found that higher TRFATP and lower LFATP were associated with higher CVD risk.

In an opinion survey^71^, using DXA body measurement and machine learning methods, researchers depicted that TRFAT correlates with both mean systolic and diastolic pressure -the same as our findings. The authors have not provided trunk lean body mass results but declare that total lean body mass positively correlates with mean systolic blood pressure. In general, evidence is lacking about the association between TRFFM and hypertension risk.

### Appendicular FATP and FFM

There are conflicting data about arm and leg fat association with HTN. In a study of 3130 Chinese participants by Ye et al.^60^, fat mass percentage and lean body mass, especially in the arm, were positively associated with increased blood pressure. Nevertheless, leg lean mass showed no significant association with systolic and diastolic pressure. In another study^74^ on 399 participants, authors showed that: (1) arm fat was a positive predictor for blood pressure, (2) after full adjustment, loss of lean leg mass directly correlated with reductions in systolic blood pressure, (3) loss of leg fat and lean mass had direct beneficial changes in markers of CVD risk. More conflicting results exist: positive association of mid-upper arm circumference with increased hypertension risk^75^, and significant inverse association between the leg and arm total fat percentage with hypertension^76^.

The exact mechanism by which LFATP and LFFM modulate blood pressure is still unclear. Regional fat deposition in the legs, mainly subcutaneous, reduces fatty acid turnover and downregulates triglyceride production in the blood. Therefore, it acts as a “metabolic sink” and preserves other tissues from lipotoxicity, protects endothelium against damage, and maintains elasticity and compliance of arterioles^74,77^. Another possible mechanism is that as subcutaneous fat, it may decrease the activation of renin–angiotensin–aldosterone and the sympathetic system^77^. Also, for FFM, some studies declare that muscle mass has a protective role in blood pressure^78,79^. However, Ye et al.^60^ suggest that previous studies on appendicular lean mass or skeletal muscle did not control fat mass and fat distribution in their analysis, leading to inaccurate results.

### Gender and age

Sex differences did not predict hypertension in our study population; however, the association was negative in males and positive in females. Previous studies showed that in men, lower body fat (thigh or gynoid) had a more protective effect on cardio-metabolic risks, such as elevated blood pressure. The effects of sex hormones on subcutaneous fat mass in these regions might explain this sex difference^80^.

Based on our results, age had a positive association with hypertension. Likewise, a study on the Chinese population age indicated an independent association in both men and women with hypertension^81^. However, results are not always positive; in a study performed on Brazilian children and adolescents, regardless of sex, the authors observed no significant association between age and systolic blood pressure^82^.

### BMR

Our study demonstrated a strong inverse relationship between BMR and hypertension, but this is not reported elsewhere. A study in Bangladeshi adults showed a positive relation between BMR and blood pressure, suggesting that upregulated BMR may elevate blood pressure by accelerating thyroid hormone levels and increasing sympathetic tone and oxidative damage^83^. Further investigation is required.

### Strengths and limitations

The implication of machine learning in a cohort of patients is the main strength of our study. Machine learning methods are more precise than traditional ones, so we believe that our findings can resolve the conflicting results regarding our research question. Nevertheless, this study has some limitations including lack of data about the use of anti-hypertensive drugs and other anthropometric indices such as waist circumference. Also, BIA of TRFAT do not differentiate between visceral and subcutaneous abdominal adipose tissues. However, we aimed to use an available method for body composition analysis and BIA is a simple, safe, and readily available method –unlike DEXA, CT scan, and MRI. We suggest that future prospective studies use machine learning methods and body composition analyses to predict hypertension among different ethnic groups. In addition, this study can be extended to more clinical samples. Consequently, classification methods especially the autoMLP are expected to have better performance.

## Conclusion

Given that body fat and its distribution are risk factors for hypertension, we used machine learning methods to study these relations. With an acceptable accuracy, we confirmed that BIA-derived body composition predicts hypertension. Also, total and regional FATP, higher age, and female gender had a positive relation with hypertension while it was the exact contrary for total and regional FFM, BMR, and male gender.

## Data Availability

Data are available from the authors upon reasonable request from the corresponding author, Hamed Bazrafshan Drissi.
